# Using a hybrid neuron in physiologically inspired models of the basal ganglia

**DOI:** 10.3389/fncom.2013.00088

**Published:** 2013-07-05

**Authors:** Corey M. Thibeault, Narayan Srinivasa

**Affiliations:** ^1^Center for Neural and Emergent Systems, Information and System Sciences Laboratory, HRL Laboratories LLC.Malibu, CA, USA; ^2^Department of Electrical and Biomedical Engineering, The University of NevadaReno, NV, USA; ^3^Department of Computer Science and Engineering, The University of NevadaReno, NV, USA

**Keywords:** basal ganglia models, Izhikevich neuron, Parkinson's disease, deep brain stimulation, action-selection, correlation analysis

## Abstract

Our current understanding of the basal ganglia (BG) has facilitated the creation of computational models that have contributed novel theories, explored new functional anatomy and demonstrated results complementing physiological experiments. However, the utility of these models extends beyond these applications. Particularly in neuromorphic engineering, where the basal ganglia's role in computation is important for applications such as power efficient autonomous agents and model-based control strategies. The neurons used in existing computational models of the BG, however, are not amenable for many low-power hardware implementations. Motivated by a need for more hardware accessible networks, we replicate four published models of the BG, spanning single neuron and small networks, replacing the more computationally expensive neuron models with an Izhikevich hybrid neuron. This begins with a network modeling action-selection, where the basal activity levels and the ability to appropriately select the most salient input is reproduced. A Parkinson's disease model is then explored under normal conditions, Parkinsonian conditions and during subthalamic nucleus deep brain stimulation (DBS). The resulting network is capable of replicating the loss of thalamic relay capabilities in the Parkinsonian state and its return under DBS. This is also demonstrated using a network capable of action-selection. Finally, a study of correlation transfer under different patterns of Parkinsonian activity is presented. These networks successfully captured the significant results of the originals studies. This not only creates a foundation for neuromorphic hardware implementations but may also support the development of large-scale biophysical models. The former potentially providing a way of improving the efficacy of DBS and the latter allowing for the efficient simulation of larger more comprehensive networks.

## 1. Introduction

The basal ganglia (BG) is a primal structure spanning the telencephalic and mesencephalic regions of the nervous system. This sub-cortical structure plays a role in a number of cognitive and behavioral phenomena that include action-selection, action-gating, timing, reinforcement-learning, working memory, fatigue, apathy, goal-oriented behavior, and movement preparation. In addition, it is the epicenter of a number of neurological disorders that include Parkinson's disease and Huntington's disease as well as psychiatric disorders such as schizophrenia and obsessive compulsive behavior.

Computational models of the BG have proved useful in many aspects of neuroscience; including developing novel theories of Parkinson's disease and deep brain stimulation (DBS) (Rubin and Terman, 2004) or testing novel functional anatomy involved in action-selection (Gurney et al., 2001). Given its prominent role in behavioral function as well as its clinical relevance, models of the BG are important to many different aspects of neuroscience application and research. One of particular importance to this work, is neuromorphic engineering.

Neuromorphic engineering is a bottom–up approach to neural modeling where the single neuron dynamics are implemented in hardware specific digital and analog circuits. The neurons are then connected to each other through different levels of communication fabric to create large neural simulations. These low-power application specific options offer not only a mechanism for simulating large-scale neural models but also a means of embodying them in mobile agents. First introduced by Mead (1989), modern manufacturing processes with higher yield and transistor density have resulted in a renaissance for neuromorphic engineering. This is evidenced by a number of projects such as FACETS/BrainScaleS (Schemmel et al., 2010), SpiNNaker (Furber et al., 2012), Neurogrid (Gao et al., 2012), and SyNAPSE (Merolla et al., 2011; Srinivasa and Cruz-Albrecht, 2012) to name a few. Each of these have different methods of simulating and abstracting models of the nervous system. However, they share the common goal of creating large-scale models of the nervous system.

One possible application for these low-power neuromorphic processors is in neural control engineering. The work of Voss et al. (2004) demonstrated one of the first examples of combining dynamical control theory and electrophysiology where the state of a reduced neuron model was estimated using an unscented Kalman filter. This helped establish the strategies for observing and controlling the highly non-linear dynamics of neural systems. Although that original application contained only a single neuron and merely estimated the missing model parameters, subsequent work has shown the robustness of this strategy in a number of control and estimation paradigms (Abarbanel et al., 2008; Ullah and Schiff, 2009, 2010; Schiff, 2010, 2012; Aprasoff and Donchin, 2012).

Closed-loop control of DBS has been demonstrated as a potentially more effective treatment of Parkinson's disease than open-loop configurations (Rosin et al., 2011). Model-based control strategies may provide further clinical benefit as well as improved power efficiency. A key component to power-efficiency may lie in the neuromorphic hardware discussed above. Before these theories can be put into practice the biophysical models of the BG need to be further developed (Modolo et al., 2012). In addition, more hardware friendly versions of those computational models need to be explored.

The individual neurons that comprise the subcortical structures of the BG have distinct firing characteristics that are thought to be essential for its function. These firing patterns are too complex for simple neuron models such as the leaky-integrate-and-fire (LIF) (Dayan and Abbott, 2005) and the more detailed conductance based models are difficult to implement in hardware (Rangan et al., 2010). In order to satisfy the firing requirements and facilitate the realization of these models in hardware we are proposing the use of the simple hybrid neuron of Izhikevich (2003).

Simultaneously lauded for its ability to replicate a multitude of neuronal dynamics (Izhikevich, 2007) and criticized for its lack of stability (Touboul, 2009), the Izhikevich simple hybrid neuron appears to be an ideal candidate for large-scale biologically realistic models of the BG. However, its use in existing spiking models has been sparse. The model presented by Igarashi et al. (2011) utilized the hybrid model but only for neurons in the striatum. The other nuclei are modeled using a conductance-based integrate-and-fire model. Modolo et al. (2007a,b) incorporated the simple Izhikevich neuron into a population based model. However, the neuron parameters were selected to achieve single neuron dynamics within the tonic regime described by Izhikevich (2003). Similarly, Modolo et al. (2008) and Modolo and Beuter (2009) explored a population-based model of the STN-GPe complex that was also based on the simple hybrid neuron. In that work, rather than just using the tonic regime, the firing dynamics for both nuclei were replicated. Latteri et al. (2011) explored the synchronization characteristics of a population of coupled neurons. A single type of Izhikevich model was used with the simulations matching both experimental results and model results based on the Morris-Lecar neuron model.

In this paper we expand the BG computational modeling landscape by recreating four published models of the BG using the hybrid neuron. The motivation is to justify its use in developing novel BG theories as well as its inclusion in future neuromorphic hardware. The studies, outlined below, were selected based on biological fidelity, functional significance, and level of abstraction.

The network model of Humphries et al. (2006) simulated the BG's ability to resolve multiple competing signals using a physiologically realistic neuron model. It was the first computational model of action-selection to include experimentally observed firing properties of the BG neurons. The result of this effort was a network that successfully demonstrated action-selection and replicated the results of several experimental studies. This same network was later used to study subthalamic DBS in Parkinson's disease (Humphries and Gurney, 2012).

While that network consisted of 960 neurons the BG network of Rubin and Terman (2004) demonstrated the efficacy of subthalamic DBS with only 50. The simplicity of the network model combined with the comprehensive mathematical analysis has made it a seminal work in BG modeling. The study emphasized the BG's influence on the thalamus, how that is lost in Parkinson's disease and the paradoxical mechanism of action of DBS in returning that influence.

The network model was later extended in the study presented by Pirini et al. (2009). In that, the effects of DBS were framed in the context of action-selection of two BG channels. The simulations supported the conclusions of Rubin and Terman (2004) by demonstrating similar results using an additional task.

The effects of correlated firing observed in Parkinson's disease at the single neuron level were presented in the study of Reitsma et al. (2011). Three different patterns of firing observed experimentally in the Parkinsonian BG were simulated and the results illustrate how these pathological cases can affect BG performance. Exploring single cell responses to correlated inputs is important in understanding how population level effects translate down to single cells. In addition, this helps in the elucidation of the important properties of correlations (Cohen and Kohn, 2011).

These represent different but complementary approaches to capturing the dynamics of the BG. The focus of this paper is on faithfully replicating both the single neuron and overall network dynamics while staying as close to the original architectures as possible. Establishing the utility of the hybrid neuron at these varying levels of detail creates a foundation for network models amenable to hardware implementations.

## 2. Materials and methods

### 2.1. Simple Izhikevich neuron

Hybrid neuron models are characterized by a set of continuous non-linear spike functions and a discontinuous after-spike reset. These models are derived from dynamical system theory and are capable of replicating the firing activity of many cortical neurons (Izhikevich, 2007). The model is expressed by the simple membrane voltage equation,

(1) $$\frac{dV}{dt}=0.04V^{2}+5V+140-u+I,$$

a recovery variable,

(2) $$\frac{du}{dt}=a(bV-u),$$

and the spike reset equations

$$\text{if V}\geq \text{30, then}\{\begin{array}{l} V\leftarrow c\\ u\leftarrow u+d. \end{array}$$

The current *I* represents the sum of the total synaptic and externally applied currents. The synaptic influence on the cell is defined by

(3) $$I_{\text{syn}}=g_{\text{syn}}\cdot (E_{\text{syn}}-V),$$

where *g*_syn_ is the synaptic conductance and *E*_syn_ is the reversal potential of the synapse. After the arrival of a spike the synaptic currents are decayed based on

(4) $$\tau_{\text{syn}}\frac{dg_{i}^{\text{syn}}}{dt}=-g_{i}^{\text{syn}}+\sum W_{ji}\delta \left(t-t_{j}\right),$$

where *g*^syn^_*i*_ is the synaptic conductance for neuron *i* and syn ∈ {E, I}, for excitatory and inhibitory synapses, respectively. In all of the simulations presented here a Euler integration method is used with time step τ = 1 ms.

### 2.2. Modeling Basal Ganglia nuclei

There are six nuclei in the classic model of the BG: the Striatum, the external segment of the Globus Pallidus (GPe), the internal segment of the Globus Pallidus (GPi), the Subthalmic Nucleus (STN), the Substania Nigra pars compacta (SNc), and the Substania Nigra pars reticulata (SNr) (Obeso and Lanciego, 2011). The major input into these nuclei come from a large number of cortical areas. In fact, almost every layered neocortical region contain outgoing connections from layer V into the striatum of the BG (Bolam et al., 2000; Gerfen and Bolam, 2010). The output connections are split mainly between the brainstem and thalamus.

#### 2.2.1. Dual pathways

There is a separation of the functional anatomy of the BG nuclei that forms parallel paths through the organ. These paths have been defined as the “direct” pathway, so named because the input neurons directly connect to the output nuclei, and the “indirect” pathway, named as such due to the elongated path through the inner BG nuclei. The functional significance of these paths is still debated, however, their existence is not. Historically the pathways have been functionally separated as the “go” (direct) and the “no-go” (indirect) pathways (O'Reilly, 2006; Cohen and Frank, 2009; Shouno et al., 2009; Chakravarthy et al., 2010; Krishnan et al., 2011). These dual pathways play a role in the action-selection models presented here. However, using the Humphries et al. (2006) terminology the direct pathway acts as the “Selection” mechanism and the indirect pathway as the “Control” mechanism.

#### 2.2.2. Striatum

In primates the striatum can be separated into two functional regions, the caudate and the putamen. The caudate is primarily innervated by prefrontal cortical connections. While the putamen receives afferents preferentially from the motor and somatosensory regions (Gerfen and Bolam, 2010), they are often included as a single unit since there no clear demarcation between the two. In addition, there are overlaps in cortical input to the putamen (Gerfen and Bolam, 2010).

The recipients of the cortical inputs are medium-size spiny GABAergic neurons that comprise about 95% of the striatum (Oorschot, 2010). These projection neurons are separated based on their subcortical targets. The “direct” pathway neurons directly innervate the output nuclei of the BG. Those neurons that comprise the “indirect” pathway connect to the intermediate structures as described above. The remaining 5% of neurons are interneurons that do not project beyond the boundaries of the striatum.

**Figure 1:**
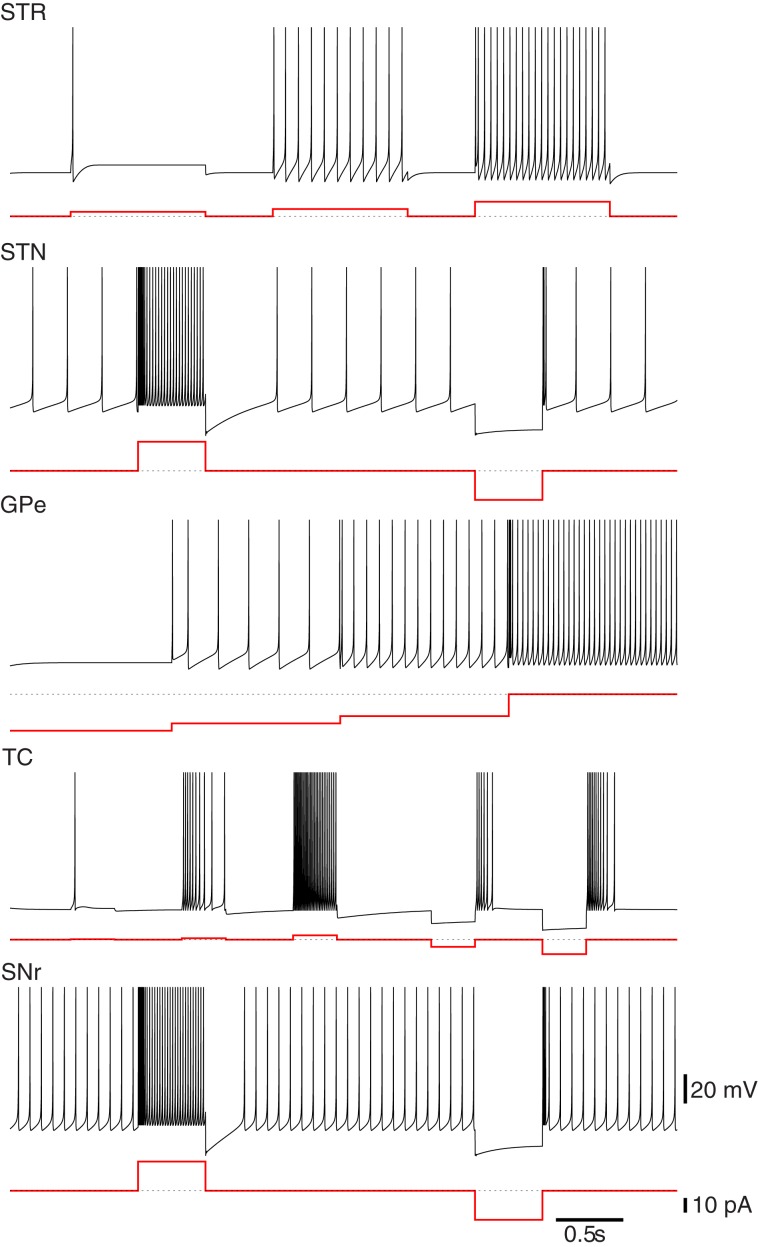
**Firing characteristics of single model neurons of the Basal Ganglia**. Many of the firing characteristics inherent to neurons of the BG nuclei are captured by the simple hybrid model. The model parameters used to achieve these patterns are Striatum (STR): (*a* = 0.02, *b* = 0.2, *c* = −65.0, *d* = 8.0), Subthalmic Nucleus (STN): (*a* = 0.005, *b* = 0.265, *c* = −65.0, *d* = 2.0), Globus Pallidus Externa (GPe): (*a* = 0.005, *b* = 0.585, *c* = −65.0, *d* = 4.0), Thalamocortical Neuron (TC): (*a* = 0.002, *b* = 0.25, *c* = −65.0, *d* = 0.05), Substania Nigra pars reticulata (SNr): (*a* = 0.005, *b* = 0.32, *c* = −65.0, *d* = 2.0). Note that the globus pallidus interna (GPi) response is not shown here but has similar firing characteristics to the GPe neurons only with a higher basal level of firing.

For the networks described in this work, we selected parameters that matched the medium-size spiny neurons of the striatum. The model neuron in Figure 1 responds to increases in depolarizing inputs with an increase in firing rate. Notably absent are the presence of “up” and “down” states observed *in vivo*. This bistability contributes to the response to depolarizing currents and in particular, the long-latency spike-discharges. The simple hybrid model used here is not capable of replicating those dynamics, although there are versions of it that can (Izhikevich, 2007; Humphries et al., 2009). Despite this, the model is still suitable as the bistablity phenomena is unnecessary for the networks replicated here and may not exist *in vitro* (Humphries et al., 2006).

#### 2.2.3. Globus Pallidus externa

The external segment of the Globus Pallidus is considered part of the indirect pathway and is composed mainly of spontaneously active inhibitory neurons that utilize GABA for neurotransmission. In the traditional BG models the inputs into the GPe are GABAergic inhibitory connections from striatum as well as glutamaterigc excitatory inputs from the subthalamic nucleus. The major output targets are a feedback connection to the subthalamic nucleus and a forward connection to the internal segment of the globus pallidus. However, in addition to these the GPe also contains projections to the substania nigra and back to the striatum.

The GPe neuron model presented in Figure 1 is intrinsically active and responds to hyperpolarizations with a decrease in tonic firing. Unlike the neuron models of Rubin and Terman (2004) the simple hybrid model is unable to replicate the transition from tonic firing to bursting in response to sufficient hyperpolarizations. This however, did not appear to be a necessary property to replicate the dynamics of the model.

#### 2.2.4. Globus Pallidus interna

The internal segment of the Globus Pallidus is one of the major output nuclei of the BG. This area contains mostly inhibitory neurons with a high level of basal activity (Humphries et al., 2006). The major input connections are inhibitory innervations from the GPe and the striatum as well as excitatory innervations from the subthalamic nucleus. The GPi innervates the thalamus and among other things is involved in limb and trunk movements. The firing patterns of the GPi neurons employed here match those of the GPe neurons presented above (not shown). They do however, have a higher level of basal activity (Rubin and Terman, 2004).

#### 2.2.5. Subthalamic nucleus

The Subthalamis Nucleus appears to contain only one type of neuron that is excitatory and releases glutamate (Gerfen and Bolam, 2010). Input into the STN arise from the GPe but also directly from the cortex. The latter innervations have been labeled by some as the “hyperdirect” pathway since this avoids the striatum directly.

The neurons of the STN model used here have spontaneous activity of around 5–10 Hz. Physiologically this is due to voltage activated *Na*^+^ channels. In addition, when a depolarizing current is applied the STN model responds with a high-frequency tonic firing and a quiescent period after sustained depolarization. The model will fire rebound bursts in response to sufficient hyperpolarizations (see Figure 1). Missing from this model are the spontaneous bursts in the absence of inputs as well as plateau deploarizations as observed experimentally. The simple hybrid model is incapable of including all of the STN cell dynamics.

#### 2.2.6. Substania Nigra pars compacta

Included here only for completeness, the Substania Nigra pars compacta is at the core of the dopaminergic system of the midbrain. These neurons are spontaneously active and provide tonic and phasic releases of dopamine at about 5 Hz (Cohen and Frank, 2009). The neurons of the SNc are densely connected and principally output to the patch/matrix layout of the striatum. The ventral region of the SNc connects to small islands or patches spatially segregated in the striatum. While the neurons of the dorsal SNc project to the regions surrounding the patches, referred to as the matrix (Gerfen and Bolam, 2010). The functional implications of this organization are still unknown.

#### 2.2.7. Substania Nigra pars reticulata

The Substania Nigra pars reticulata is the other output nuclei of the BG and is responsible for head, neck and eye movements. The SNr is comprised mainly of GABAergic inhibitory neurons and similar to the GPi has a high basal level of activity. The SNr receives inputs from STN and striatum and outputs to the superior colliculus, the thalamus and the pedunculopontine nucleus. Figure 1 presents the single neuron dynamics of the SNr used here.

#### 2.2.8. Thalamus

It is theorized that the primary role of the thalamus is to modulate and process the information entering the cortex (Sherman and Guillery, 2002). A thalamocortical relay neuron is used here to model that influence. These are bimodal neurons that alternate between a tonic firing mode and a burst firing mode depending on the voltage and time dependent *CA*^2+^ T-current (Sherman, 2001).

Parameters were selected to achieve the dual firing modes described in Sherman (2001). They do not fire spontaneously but when in the tonic mode show an increase in firing rate in response to larger depolarizing currents. In the burst mode, when subjected to sustained hyperpolarizing input, the model neurons respond with periods of bursting that is dependent on the strength and duration of the applied current (see Figure 1).

### 2.3. A physiologically plausible model of action-selection

Conceptually, action-selection is the arbitration of competing signals and the role of the BG is to select the most appropriate one. The complex circuitry of the BG is active in gating information flow in the frontal cortex and the selection mechanism can affect simple action all the way up to behaviors and cognitive processing (Cohen and Frank, 2009). To explore that mechanism in a physiologically meaningful way, Humphries et al. (2006) connected populations of realistic “spiking neurons” configured using the functional anatomy of Gurney et al. (2001). The biological fidelity of the model was validated at the population level as well as single-unit recordings from networks replicating anesthetized or lesioned conditions. This was the first network model recreated here.

**Figure 2:**
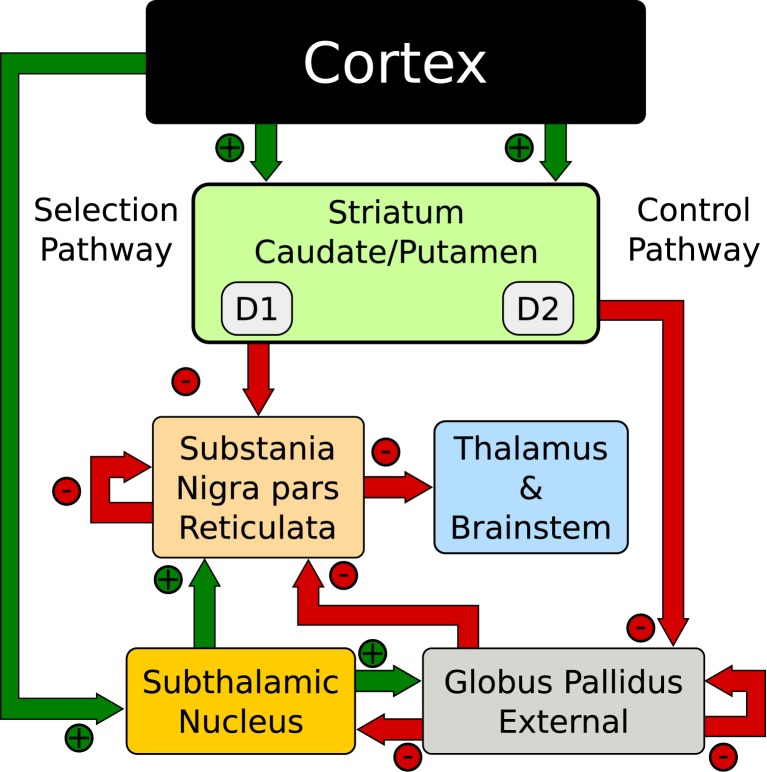
**Action-selection network model (Humphries et al., 2006)**.

The network exploits the concept of competing anatomical channels within the BG. Three separate channels were constructed using the layout of Figure 2. Each population consisted of 64 neurons per channel with the parameters of Table 1A. Most connections of the model were focused projections where post-synaptic connections were randomly sampled within a channel using the probability ρ_*c*_ = 0.25. However, the diffuse projections listed in Table 1B, spanned all channels and the connection probability ρ_*c*_ was divided among each of those. This is consistent with the more diffuse outputs from the STN (Haber, 2010). Cortical inputs to the striatum were simulated as Poisson random spikes. The synaptic parameters are listed in Table 1C.

Table 1**Parameters for the model of action-selection**.

**Table:** 

**Neural region**	***a***	***b***	***c***	***d***	**I**_**app**_ **(pA)**
**A. NEURON PARAMETERS**					
STR (D1/D2)	0.02	0.2	−65.0	8.0	0.0
SNr	0.005	0.320	−65.0	2.0	25.0
STN	0.005	0.265	−65.0	2.0	20.0
GPe	0.005	0.585	−65.0	4.0	5.0

**Table:** 

**Source → destination**	**Synaptic conductance**	**Delay (ms)**
**B. CONNECTIONS**		
Cortical input → STR	0.2	11
Cortical input → STN	0.2	6
Striatum D1 → SNr	0.12	6
Striatum D2 → GPe	0.1	6
GPe → STN	0.025	6
GPe → GPe	0.025	2
GPe → SNr	0.015	6
SNr → SNr	0.015	6
STN → SNr focused	0.075	2
STN → SNr diffuse	0.35	2
STN → GPe focused	0.075	2
STN → GPe diffuse	0.35	2

**Table:** 

**Parameter**	**Value**
**C. SYNAPTIC PARAMETERS**	
τ_ge_	5 (ms)
τ_gi_	100 (ms)
*E*_exc_	0 (mV)
*E*_inh_	−80 (mV)
*V*_rest_	0 (mV)

### 2.4. The parkinsonian BG and deep brain stimulation

The modeling study of Rubin and Terman (2004) presented a network level explanation for the mechanism of action of DBS. In Parkinson's disease there is a marked loss of dopaminergic cells in the SNc. The reduction in tonic and phasic dopamine onto the BG nuclei results in, among many other phenomena, a rhythmic synchronization of the major output nuclei of the BG. Within the computational model this resulted in a decrease in the ability of thalamocortical neurons to respond to depolarizing cortical inputs. It was hypothesized that this loss of relay fidelity is one of the underlying causes for many clinical Parkinsonian symptoms.

**Figure 3:**
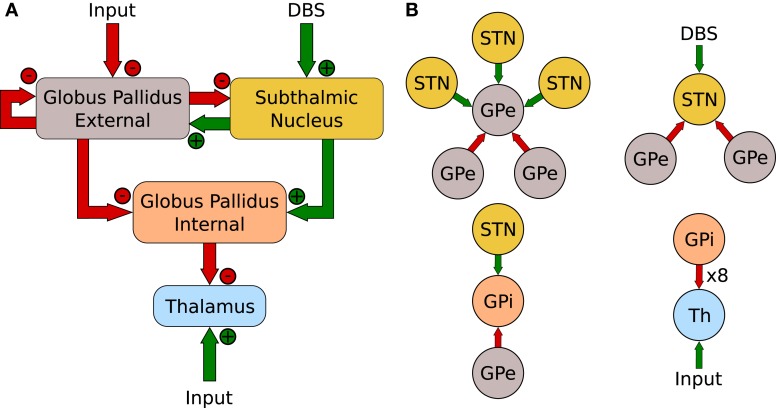
**(A)** Network layout of Rubin and Terman (2004). **(B)** Individual neuron connections.

The network, referred to as the RT Model, is illustrated in Figure 3A. It consists of four populations: the GPe, STN, GPi and thalamus. With the exception of the thalamus, that contains 2 neurons, each population has 16 neurons. Unlike the action-selection model presented above, the RT model maintained consistent network connectivity that was exactly the same used by Rubin and Terman (2004). Figure 3B illustrates the connectivity patterns for individual neurons of the network.

The parameters used for the neurons of the RT model are listed in Table 2A. Note that similar to the original model a constant input current, *I*_app_, is added to each of the neurons to increase the basal activity. The synaptic conductances were randomly selected from a normal distribution with the ranges specified in Table 2B. There are two sources of depolarizing input current used in this model. Both follow an equation of the form

(5) $$I=i_{\chi }\cdot H\left(\text{sin}\frac{2\pi t}{\rho_{\chi }}\right)\cdot \left(1-H\left(\text{sin}\frac{2\pi (t+\delta_{\chi })}{\rho_{\chi }}\right)\right),$$

where *H* is the Heaviside function and χ ∈ {SM, DBS}, SM and DBS, are somatomotor and DBS, respectively. The values used for each of these currents is presented in Table 2C. The synaptic delay, imposed by the simulator, was 2 ms. The synaptic parameters matched those in Table 1C.

Table 2**Model parameters for Parkinson's disease model**.

**Table:** 

**Neural region**	***a***	***b***	***c***	***d***	**I**_**app**_ **(pA)**
**A. NEURON PARAMETERS**					
GPe	0.005	0.585	−65.0	4.0	5.0
GPi	0.005	1.2	−65.0	4.0	7.0
STN	0.005	0.265	−65.0	2.0	15.0
TC	0.002	0.25	−65.0	0.05	0.0

**Table:** 

**Source → destination**	**Synaptic conductance range**	
	**Low**	**High**
**B. CONNECTIONS**		
GPe → GPe	0.1	0.2
GPe → STN	0.1	0.2
GPe → GPi	0.3	0.4
STN → GPe	0.2	0.3
STN → GPi	0.5	0.6
GPi → TC	0.02	0.0225

**Table:** 

**Parameter**	**Value**
**C. INPUT PARAMETERS**	
*i*_SM_	30 (pA)
δ_SM_	3 (ms)
ρ_SM_	25 (ms)
*i*_DBS_	130 (pA)
δ_DBS_	1 (ms)
ρ_DBS_	8 (ms)

The role of the STN and GPe in this model is to create the patterns of activity within the GPi that are observed experimentally. As discussed above, the GPi is the major output nuclei and is responsible here for appropriately controlling the activity of the thalamic neurons. The role of the thalamus in this case is simplified into a relay station; responsible for appropriately relaying depolarizing signals from somatomotor inputs.

Under the normal mode of operation the nuclei of the BG produce irregular firing patterns and the thalamus is capable of relaying somatomotor information reliably. In the Parkinsonian state the GPe and STN nuclei have more regular synchronized firing rates and the thalamic relay fidelity is greatly diminished. Similar to the original work the Parkinsonian mode is accomplished by reducing GPe → GPe to 0 as well as reducing the current *I*_app_ to −19. This follows the procedure of Rubin and Terman (2004) and is based on the activity patterns of Terman et al. (2002). Finally, the application of DBS to the STN is used to restore relay capabilities while in the Parkinsonian state.

To quantitatively evaluate the performance of the model in each of the three states, an error index measure was introduced in Rubin and Terman (2004). This is defined as

(6) $$EI=\frac{m+e}{t},$$

where *m*, representing misses, is the number of somatomotor signals that were not relayed, *e*, erroneous, is the number of responses that result in multiple spikes and *t* is the total number of stimulus inputs. The error index evaluation was completed by running 20 simulations of the model in each of the modes described above. Each run resulted in different results due to the randomly selected connection weights described in Table 2B. The error index was calculated for each of the TC cells and a box and whisker plot were created to compare with Rubin and Terman (2004).

### 2.5. Restoring action-selection in the parkinsonian basal ganglia

In addition to exploring different sites of DBS application, the work of Pirini et al. (2009) divided the RT model into 2 distinct control channels and added a striatal current into the GPi, representing the direct pathway discussed above. The model was capable of demonstrating simple two channel action-selection by way of disinhibition. The successful switching between channels was lost under Parkinsonian conditions but could be restored by the application of DBS into the STN.

The exact two channel network from Pirini et al. (2009) was constructed here. The network layout and individual neuron connections matched those of Figure 3 and the parameters of Table 2 were used with some modifications to handle the change in network configuration as well as the stochastic input pattern. The somatomotor input into the TC cells was reduced to 24.5 pA and the duration, δ_SM_ was changed to 4 ms. In addition, the pulse times were randomly selected from an exponential distribution with a mean of 15 Hz. The DBS current was reduced to 50 pA and the period ρ_DBS_ was reduced to 7 ms to more closely match the original work.

The action-selection mechanism signaled by the striatum is modeled as a current input into the GPi nucleus. Under normal conditions the current values are *I*_On_ = 0 pA and *I*_Off_ = 27 pA. For the Parkinsonian condition the values are set to *I*_On_ = 0 pA and *I*_Off_ = 22 pA to represent the loss of striatal inputs into the GPi. Each state lasts 2 s before switching.

### 2.6. Thalamic relay fidelity between the BG and thalamus

Correlated firing in neuronal ensembles is important in both understanding information encoding and in interpreting functional anatomy (Cohen and Kohn, 2011). Correlated activity in many brain regions has been linked to stimulus decoding and discrimination, attention, and motor behavior (de la Rocha et al., 2007). In addition, highly correlated firing has been associated with pathological conditions. In the BG in particular, correlated activity of globus pallidus internal (GPi) neurons is associated with Parkinson's disease or pharmacological agents causing Parkinsonian like conditions (Reitsma et al., 2011).

Reitsma et al. (2011) explored the implications the temporal relationships that emerge from the GPi have on the relay fidelity of the thalamic neurons they innervate. In the Parkinsonian BG the firing patterns become increasingly oscillatory with pronounced bursting. This synchronous fire rate can have deleterious effects on the functionality of the BG. The consequence of those patterns of activity on thalamocortical relay fidelity was explored through correlation analysis of a computational model.

One conclusion of that work was that the integrate-and-fire-or-burst (IFB) neuron model demonstrated similar firing patterns and correlation transfer to that of a conductance-based model. This not only strengthened the overall conclusions of the study but also motivated the authors to suggest the IFB model as a suitable replacement for the conductance-based model in correlation studies. Here, we explore if a similar result can be accomplished with the hybrid neuron.

The IFB model achieves the bursting dynamics of TC cells through the inclusion of a T-type calcium channel. When the membrane voltage is hyperpolarized the inactivation gate of the channel begins to deinactivate. When the membrane voltage is depolarized the channel remains activated until the gate is reinactivated. Unlike the IFB model, the hybrid neuron used here does not have an explicit bursting mechanism. Instead the recovery variable is used to put the neuron with the bursting regime of the phase-portrait (Izhikevich, 2007). Reitsma et al. (2011) demonstrated that although the T-current is required to replicate the physiological spike patterns, it is not needed to demonstrate transfer of correlations. However, our goal here was to replicate both the physiological spike patterns of the thalamocortical neurons as well as the correlation transfer.

**Figure 4:**
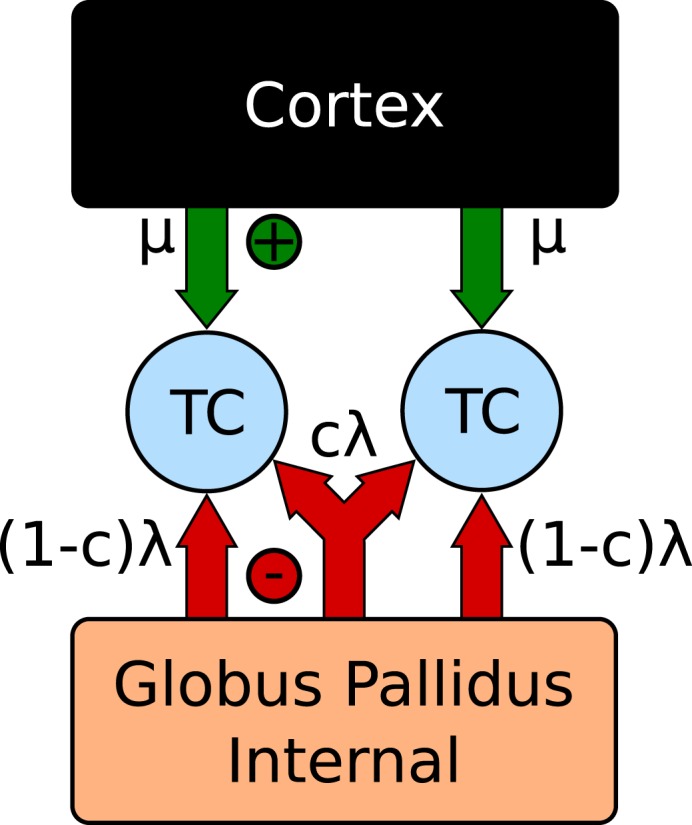
**Correlation network configuration (Reitsma et al., 2011)**.

The model consists of two spiking thalamocortical (TC) neurons subjected to inhibitory input from an engineered GPi signal as well as an excitatory input representing cortical innervations. This is illustrated in Figure 4. Each TC neuron receives independently generated 20 Hz Poisson random excitatory inputs. The GPi spike trains are generated by inhomogeneous Poisson rate functions defined as λ(*t*), with a fraction, *f*, of spikes overlapping between each TC neuron. For values of *f* > 0 a single spike train with rate λ(*t*)/*f* is constructed. Each cell then samples from this spike train with probability *f*. For *f* = 0 two Poisson random spike trains are generated using a common rate function λ(*t*).

The model and corresponding analysis was computed using the numerical programming language Octave (Eaton et al., 2008). Table 3 presents the parameters used in the model. The simple hybrid neuron of section 2.1 is used, however, to increase the stability of the simulations under the increased synaptic activity of the GPi the hybrid solution method from Izhikevich (2010) was employed. The hybrid numerical method treats the synaptic influence implicitly resulting in a linear dependence on the future value of the membrane voltage.

**Table 3:** **Parameters for the model of correlation transfer**.

**Parameter**	**Value**
*a*	0.002
*b*	0.25
*c*	−65.0
*d*	0.05
*g*_*e*_	0.12
τ_*e*_	6.0 (ms)
*V*_*e*_	0.0 (mV)
*g*_*i*_	0.09
τ_*i*_	15.0 (ms)
*V*_*i*_	−85.0 (mV)

#### 2.6.1. Input patterns

**Figure 5:**
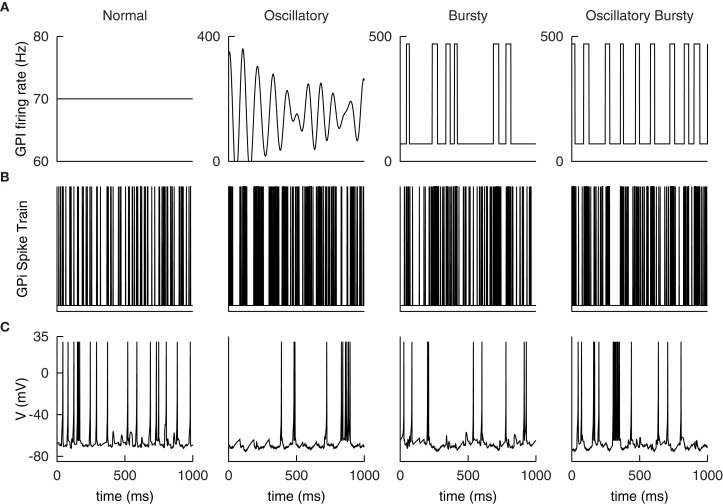
**Example GPi spike patterns and TC cell responses for each of the four modes. (A)** Example input rate functions. Resulting GPi spike trains, **(B)**, and TC Cell responses, **(C)**.

Consistent with the original work, four different GPi input patterns are constructed to emulate normal and Parkinsonian conditions observed experimentally. Samples of the rate functions are illustrated in Figure 5A. The normal input is a constant 70 Hz Poisson random spike train.

The first Parkinsonian pattern, labeled oscillatory, is constructed as a sum of 21 sine waves. The individual sine waves have frequencies ranging from 5 to 15 Hz with step changes of 0.5 Hz between them. These are weighted by a Gaussian distribution with a mean of 10 Hz and a variance of 1.5 Hz; resulting in the 10 Hz component dominating the rate function. The phase of the sine waves are then randomly shifted and summed together. The resulting function is then amplified by 50 Hz and shifted up by 150 Hz. Any negative values are set to zero. Although constructed differently than those described in Reitsma (2010); Reitsma et al. (2011), the resulting function qualitatively matches the samples presented there. In addition, the resulting functions exhibited a distinct peak at 10 Hz, see Figure 5A below, similar to the original work. The second Parkinsonian pattern, labeled Bursty, consists of a basal level of firing at 70 Hz interrupted by random bursts stepping to 470 Hz. The duration of each burst is selected from a Gaussian distribution with a mean of 30 ms and a variance of 10 ms. The time between bursts is selected from a Poisson distribution with a mean of 70 ms. The final input pattern, labeled Oscillatory Bursty, is constructed similar to the bursty case, however, the inter-burst-interval is selected from a Gaussian distribution with a mean of 30 ms and a variance of 10 ms. This results in more periodic bursts.

These rate functions are then used to generate Poisson random spike trains. Examples of these spike trains are presented in Figure 5B with the corresponding TC neuron response in Figure 5C. These patterns were selected by Reitsma et al. (2011) to replicate firing patterns and overall spike rates found in the GPi under Parkinsonian conditions.

#### 2.6.2. TC model spike response

Both interspike interval (ISI) distributions and power spectra were computed on the model TC cells for comparisons with the original work. The power spectra was computed for the TC model spike response as well as the corresponding GPi and cortical inputs using the point process multi-taper spectrum analysis from the Chronux software package (Bokil et al., 2010).

#### 2.6.3. Correlation calculations

The measure of correlation is calculated using the Pearson's correlation coefficient. This is a spike count measurement that compares the number of spikes that occur over a window of length *T* defined as

(7) $$\rho (t)=\frac{\text{cov}(n_{1}(T),n_{2}(T))}{{\left[\text{var}(n_{1}(T))\cdot \text{var}(n_{2}(T))\right]}^{1/2}},$$

where cov is the covariance, var is the variance and *n*_1_(*T*) and *n*_2_(*T*) are spike counts at window *T*.

The correlation coefficient is used to calculate the correlation susceptibility that quantifies the degree to which correlations are transferred through the model. This is computed using the equation

(8) $$\rho_{\text{out}}(T)=S(T)\rho_{\text{in}}(T)-k,$$

where ρ_in_ and ρ_out_ are the GPi input correlation coefficient and the TC output correlation coefficient, respectively.

To demonstrate how sensitive the TC neurons were to correlated input the correlation coefficients were calculated for *f* = 0, 0.25, 0.5, 0.75, 1.0 and similar to Reitsma et al. (2011) a sample bootstrap method was used to generate confidence intervals on the analysis. For each value of *f*, 30 simulations of were run for 100 s each. This resulted in 150 pairs of correlation coefficients. A straight line was then fit between the values of ρ_in_ and ρ_out_ to find the correlation susceptibility *S* based on the slope of the line. This was completed over a range of window sizes *T*. The 150 pairs were then sampled with replacement to generate a new set correlation coefficients and *S* values. This resampling was completed 1000 times to generate 98% confidence bands for each value of *T*.

### 2.7. HRLSim

With the exception of the correlation study, all of the models were simulated using the HRLSim neural simulator package (Thibeault, 2012). HRLSim is the first distributed GPGPU spiking neural simulation environment. It currently supports two different point neuron implementations, the Leaky Integrate-and-Fire (LIF) model and the simple hybrid Izhikevich model. With an emphasis on high-performance, HRLSim was developed to support the modeling efforts of the SyNAPSE project and its team members. It has also proven extremely useful as a general neural simulation environment for other studies (Srinivasa and Cho, 2012; O'Brien and Srinivasa, 2013; Srinivasa and Jiang, 2013).

## 3. Results

### 3.1. Action-selection

**Figure 6:**
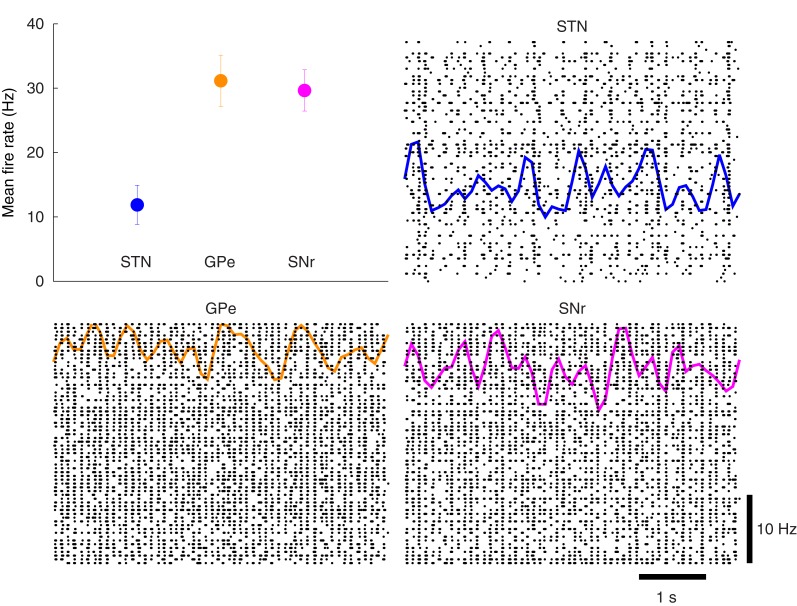
**Basal activity of the model of action-selection. Upper left:** The mean rates for the STN, GPe, and SNr qualitatively match the simulated and experimental results of Humphries et al. (2006). **Remaining plots:** The spike rasters for each of the nuclei are overlaid with the corresponding spike-count firing rates.

The action-selection model of Figure 2 was first tuned to match the original model of Humphries et al. (2006). Using the model-as-animal strategy, 15 simulations were completed with different randomly connected networks. From each of those simulations 3 cell indexes were randomly selected and the overall activity rate of the last 9 s of simulation were computed for those neurons. The mean rates and 95% confidence intervals were then computed to ensure the activity was in similar ranges to Humphries et al. (2006). This is presented in Figure 6. In addition, the spike rasters and binned spike count rate functions are included. The overall mean firing rate results are in good agreement with the original work as well as with the experimental results referenced there.

**Figure 7:**
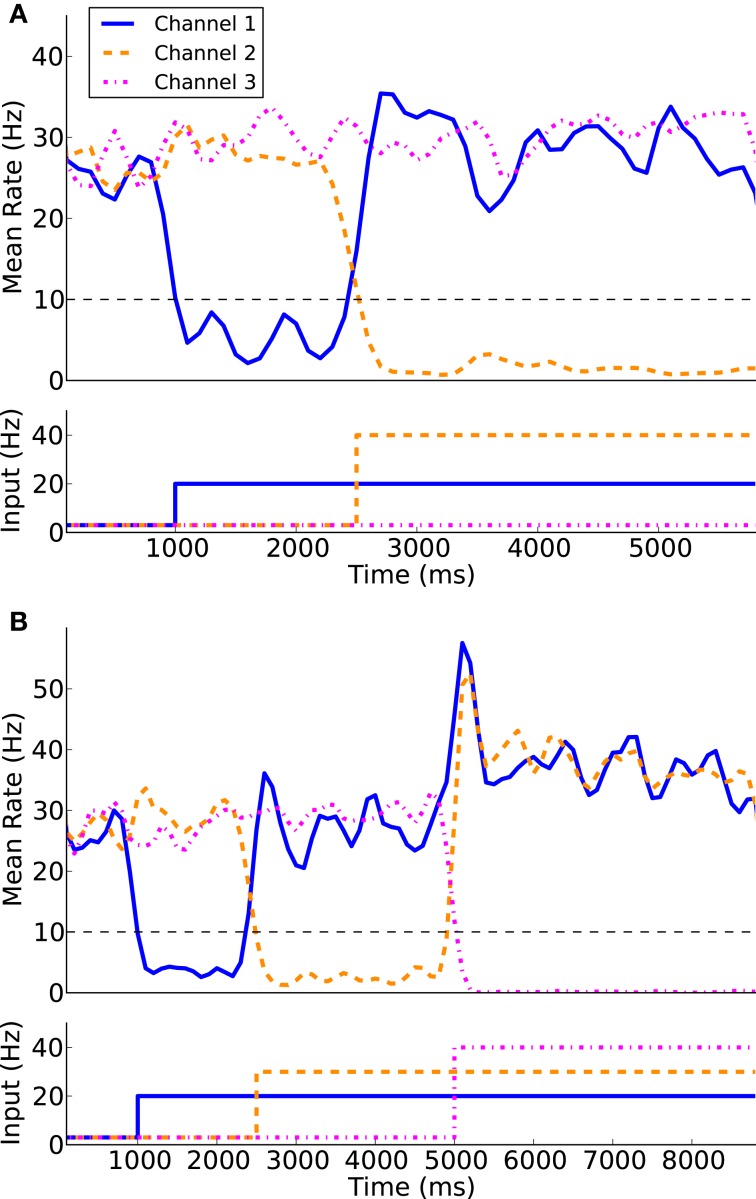
**Action-selection performance**. The model is capable of appropriate selecting the most salient input between two competing channels **(A)** as well as three competing channels **(B)**.

**Figure 8:**
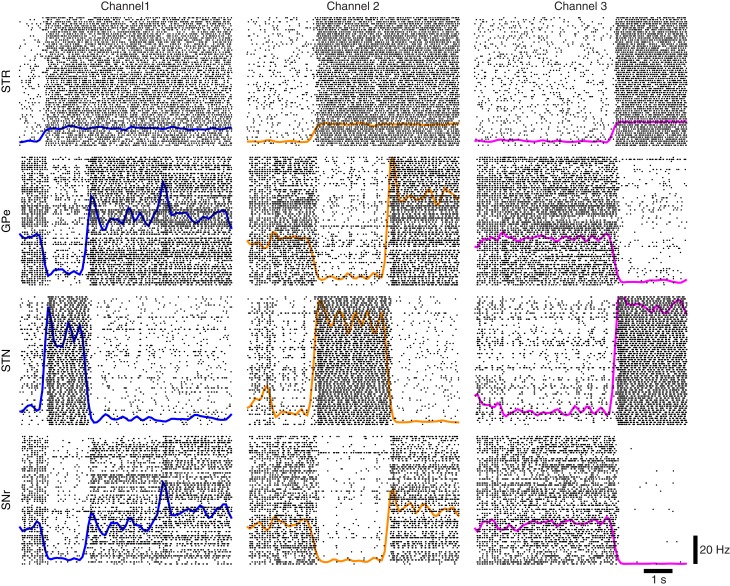
**Network response to competing inputs; spike rasters of the major nuclei of the BG action-selection with the spike count rates overlaid**.

Using the protocol of Humphries et al. (2006) for normal levels of dopamine the ability of the models to appropriately select the most salient input was first simulated using two of the three channels. Figure 7A illustrates the two channel action-selection results. Initially the network is at its basal level of activity with a 3 Hz Poisson input. At 1 s the input for channel 1 is increased to 20 Hz, causing, through disinhibition, the selection of that channel. At 2.5 s a 40 Hz cortical input is injected into Channel 2. The activity of channel 1 is pushed up to its basal level of activity and the channel 2 output is inhibited causing it to be selected. This selection mechanism is more decisive than the one presented in Humphries et al. (2006). In the original work the previously selected channel had an increase in activity that was only slightly above the selection limit. To build on this result we tested the selection capabilities of all three channels, something that was not part of the original work. The results of this are presented in Figure 7B as well as in Figure 8 where the spike rasters of the model nuclei are plotted with the overlaid spike count rate functions. This is an encouraging result and suggests that the functional anatomy of the original work can be extended to more than three channels.

### 3.2. The parkinsonian BG and deep brain stimulation

**Figure 9:**
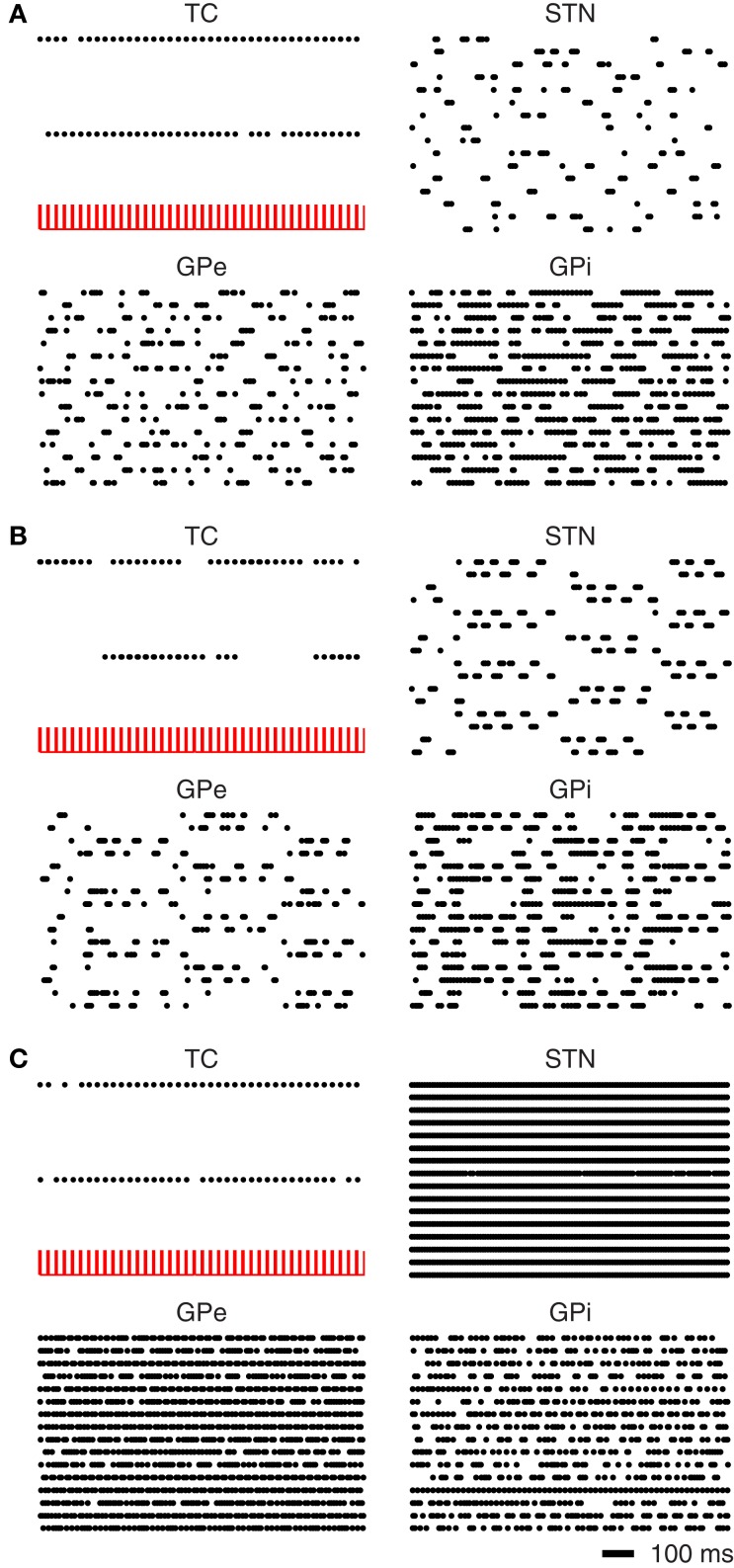
**Simulated recovery of TC relay fidelity. (A)** Under normal BG activity the thalamus is capable of relaying somatomotor inputs. **(B)** Under Parkinsonian conditions the BG nuclei fall into oscillatory firing patterns TC relay capabilities are greatly diminished. **(C)** Application of DBS to the STN restores lost TC relay fidelity.

In the normal mode the BG nuclei have irregular firing patterns with interspike interval coefficients of variation ≥1.0. With this irregular pattern of activity the thalamus is capable of reliably transmitting the somatomotor signals (see Figure 9A).

In Parkinson's disease, the firing pattern of the BG neurons have been reported to have regular synchronous firing patterns (Walters and Bergstrom, 2010). In Figure 9B it can be seen that the BG nuclei begin to fire synchronously. The neurons of the STN separate into two distinct populations with different phases of bursting. The periods of bursting oscillate around 4 Hz which is consistent with synchronous oscillations observed in the Parkinsonian BG (Walters and Bergstrom, 2010). This synchronous activity results in a marked loss of thalamic relay. As noted by Rubin and Terman (2004) the GPi activity is affected by the periods of bursting in the GPe, where the GPi would otherwise fire tonically.

The application of DBS to the STN results in an increased firing rate and a disruption of the synchronous oscillations of the BG nuclei. This disruption in the oscillatory activity is sufficient to restore the relay fidelity of the thalamus (see Figure 9C).

**Figure 10:**
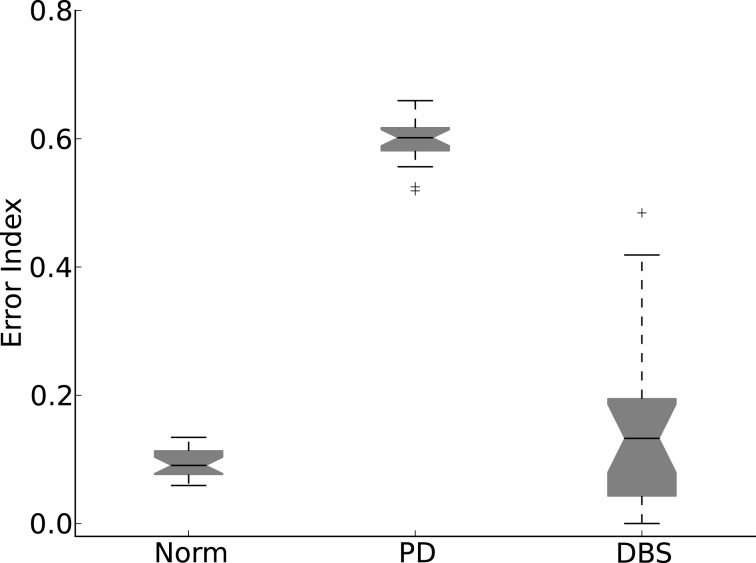
**Error index statistics**. Allowing the network connection weights to randomly change over 20 simulations results in the Normal and DBS modes operating with less errors than the PD mode.

The results of Figure 9 are quantified in Figure 10. Here the normal and DBS modes of the model result in EI medians that are comparable. Although the spreads are somewhat dissimilar, neither overlaps with the much higher values measured in the Parkinsonian state.

### 3.3. Restoring action-selection in the parkinsonian basal ganglia

**Figure 11:**
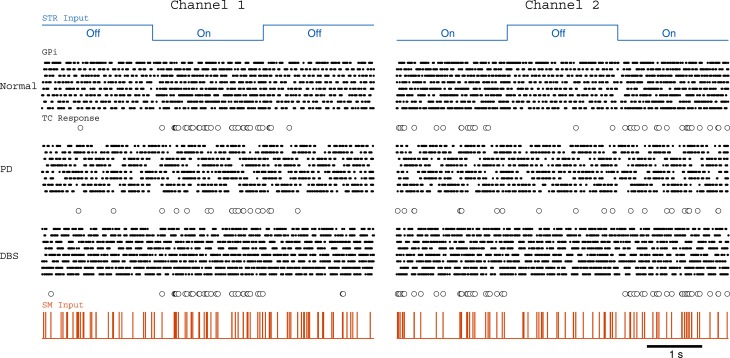
**Parkinsonian fire patterns result in a loss of accurate selection capabilities**.

The modified RT network of Pirini et al. (2009) puts the theoretical concepts of the previous sections into a dynamical model of action-selection. The results of this experiment are shown in Figure 11. Once again the loss of faithful relay can be alleviated with the application of DBS to the STN.

### 3.4. BG correlation transfer

#### 3.4.1. Firing patterns

**Figure 12:**
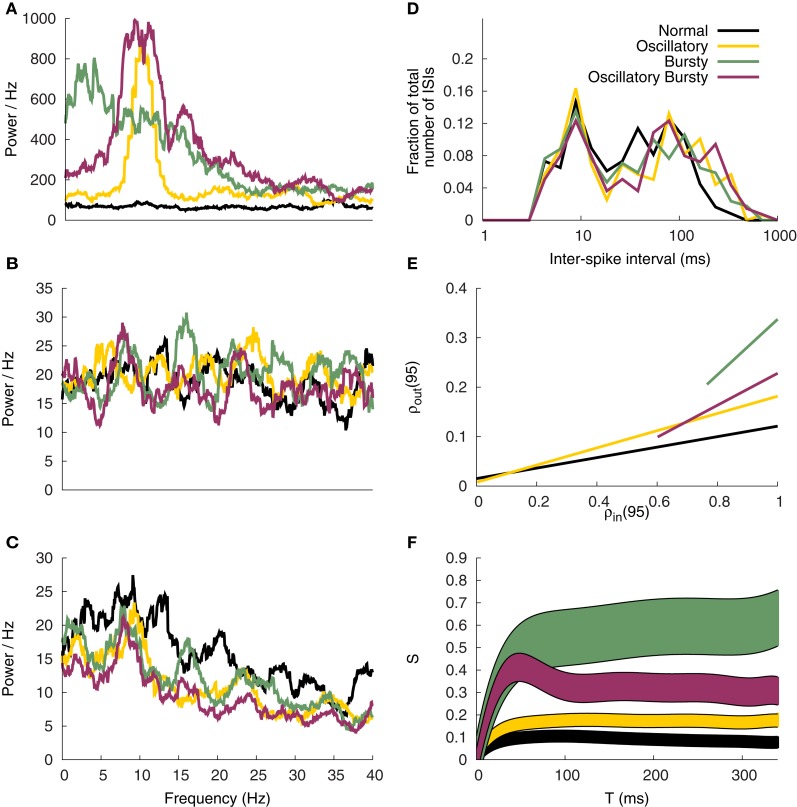
**Correlation analysis**. Spectral power of the GPi input patterns **(A)**, excitatory input **(B)** and corresponding TC cell response **(C). (D)** ISI profile of the TC Cells. **(E)** Correlation susceptibility for *T* = 95 ms **(F)** Susceptibility *S* of the TC cells based on the analysis window *T*.

Validating the generated GPi input spike trains was completed by the spectral power analysis presented in Figure 12A. As in Reitsma et al. (2011) the Oscillatory and Oscillatory Bursty patterns have clear spectral peaks at 10 Hz, while the Normal and Bursty cases have no obvious peak. As expected the cortical inputs lack a peak in the frequency range of interest (see Figure 12B).

The parameters for the model were selected based on the TC cells firing patterns and spectral analysis. Although the Normal and Bursty spectral powers do peak around 10 Hz, there are oscillations present in both (see Figure 12C). Consistent with the original work, the Oscillatory and Oscillatory Bursty cases both have more distinct peaks around 10 Hz. The discrepancies are likely due to analysis parameters and the way GPi inputs were generated, as discussed below.

There is a clear bimodality to the interspike interval histogram of Figure 12D, which is consistent with the original work. However, the first peak, at 10 ms, is lower than the 30 ms peak described by Reitsma et al. (2011). This may be a product of that model using a refractory period of 5 ms, possibly resulting in slower bursts. It may also be a product of the way the dynamical correlate of the T-current is produced in the hybrid model. This would cause the inputs to recruit the bursting regime of the model in a different or perhaps less efficient way than the IFB or conductance based models used in Reitsma et al. (2011). Despite the slight differences, the firing patterns of the hybrid model in this network are still in general agreement with Reitsma et al. (2011).

#### 3.4.2. Correlation susceptibility

The general susceptibility analysis, Figure 12F, qualitatively matches the results of Reitsma et al. (2011), however, the magnitude of the steady-state values are consistently lower than the original work. Similar results were found for our implementation of the IFB model (not presented), suggesting that the discrepancy in the magnitude of the susceptibility may arise due to differences in the way the input signals are generated. The correlation coefficients for *T* = 95 ms, Figure 12E, when fit with a linear curve illustrates the different slopes produced by the four input patterns tested. The Bursty and Oscillatory Bursty cases here have input correlation coefficients that are always greater than zero, even when *f* = 0. This is a product of generating the spike trains using a common time-dependent rate function.

In the work of Reitsma et al. (2011) the susceptibility values reach an asymptote around *T* = 200 ms. Here for the Normal and Oscillatory cases that plateau is reached much earlier, around *T* = 100 ms. The implications of this are unclear but they do not appear to affect the conclusion that the bursty inputs cause an increase in correlation susceptibility. In addition, this further supports the conclusion that the correlation results of Reitsma et al. (2011) are independent of model details. That combined with the fire pattern results above, helps validate the use of the simple hybrid model in correlation studies.

## 4. Discussion

### 4.1. Physiological model of action-selection

An interesting result of this work that was absent from Humphries et al. (2006) was the use of a neuron model that could replicate experimental dynamics at both the single neuron and population levels. Even with the added current source the LIF neuron employed by Humphries et al. (2006) is unable to completely replicate the complex fire patterns presented in Figure 1. It was argued that the most relevant dynamics are included and given that the model of Humphries et al. (2006) was able to replicate experimental results, it can be argued that the individual neuron dynamics may not be necessary. However, as illustrated by the results in Figure 7, the model presented here was able to not only selected the most salient input but also drive the activity of the previously activated channel clearly away from the selection limit. The selection results presented by Humphries et al. (2006) as well as by independent testing of the model (not presented) demonstrated a sufficient but modest increase in the activity of the previously selected channel. The increased activity of our model is large enough to push the previous channel back to its basal level of firing; reducing the possibility of selecting undesired or multiple channels. The mechanism for the improved selection capabilities is unclear and remains a focus of future studies. In addition, in the future this model will be extended to include a larger number of channels to determine how feasible it is to scale beyond the three presented here.

The original rate based model of Gurney et al. (2001) was converted into the spiking domain by Stewart et al. (2010) using LIF neurons and the Neural Engineering Framework (Eliasmith and Anderson, 2003). It was then expanded to include both action-selection and reward learning (Stewart et al., 2012). The combination of action-selection and reinforcement-learning is another aspect of this model we plan to explore.

### 4.2. The parkinsonian BG

Rubin and Terman (2004) offered one of the first models providing an explanation for the paradoxical therapeutic effects of DBS in a Parkinsonian BG. The data driven extension of this work presented by Guo et al. (2008) further supported these results and linked its theories to experimental recordings. A similar extension was performed by Meijer et al. (2011) where the relay fidelity of a single TC neuron in response to different DBS parameters was explored. Similarly, Dorval et al. (2010) used the RT model to complement human subject experiments exploring the regularity of DBS inputs.

The majority of these studies support the results of the work presented here and the theory that oscillatory inputs into the thalamus from the GPi negatively affect relay fidelity of the thalamus. In addition, constant inputs from the GPi, arising from DBS application, result in more effective relay in the thalamus (Rubin et al., 2012).

There have been a number of studies that have extended the RT model to explore the therapeutic effects of different DBS locations, protocols and strategies (Hahn and McIntyre, 2010; Guo and Rubin, 2011; Agarwal and Sarma, 2012), as well as closed loop configurations (Feng et al., 2007) and medicated states (Frank, 2005). Similarly, the inverse relationship between frequency and stimulus amplitude in clinically effective DBS has been explored with the RT Model (Cagnan et al., 2009). Similar extensions are planned for the network model presented here.

### 4.3. Thalamic relay fidelity between the BG and thalamus

The correlation study of Reitsma et al. (2011) highlighted that a number of point neuron models were capable of demonstrating how the pattern of firing in the GPi could affect correlation transfer in the thalamus. That firing patterns observed in the Parkinsonian BG result in increased correlation susceptibility of the thalamus was also found in the work presented here. This could provide an explanation for some of the pathological hallmarks of Parkinson's disease.

Although it was shown that the T-current, required for TC neuron bursting, is responsible for the spike pattern of the model, it does not appear to have an effect on the correlation transfer (Reitsma et al., 2011). Here however, we were able to demonstrate both similar spiking patterns as well as similar correlation susceptibility as the models with higher biological fidelity. These results open up a number of future studies employing the hybrid model. This includes a frequency space analysis of the correlation transfer as well as a more thorough mathematical analysis of the relationship between GPi inhibition and spike correlation.

### 4.4. BG models in neuromorphic hardware

The complexity of the neuron models explored in the original studies require a level of population specificity that is undesirable in generic hardware implementations. Although the LIF neurons of Humphries et al. (2006) are ideal for neuromorphic hardware, the gated synaptic currents as well as the piecewise calcium currents would require circuitry specific to a nuclei type and would greatly diminish the generality of the system.

The motivations for embedding BG models in hardware systems go beyond the obvious applications to intelligent agents and neurorobotics. It has been shown that the model based control concepts introduced in section 1 have a number of clinical and practical applications (Schiff, 2012). In addition to the control system computations, are the numerical calculations required for simulating the model aspect of the observer. Combining the control system with neuromorphic hardware, perhaps in a system on chip, would significantly reduce the power consumption and provide a solution appropriate for portable realization. As emphasized in Schiff (2010), even if the results of closing the loop are a reduction in battery life the model-based paradigm would be beneficial. Ideally, extended battery life will be accompanied by clinical improvements and studies cited here support the presence of both in closed-loop strategies.

**Figure 13:**
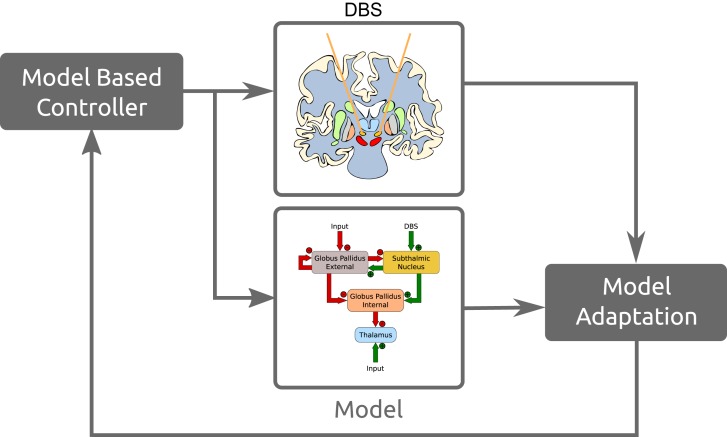
**Simplified example of how these models fit in a model based control DBS paradigm**.

Model based or model predictor control systems work as state estimators where the dynamics of the model are used to predict the state of the current system. That prediction is then corrected with new measurements. These allows us to incorporate the predictions of the system's state as well as sensor estimates with the real sensor information to get a better estimate of the actual state. Figure 13 is a simplified overview of how these models would fit into such a control system. This is a brief example of how these models and neuromorphic hardware fit in with model based control strategies, for a more extensive review see Schiff (2012).

There are a number of issues, however, beyond implementation difficulties that need to be resolved before model-based control strategies will prove useful. The level of realism required in the neuron model is still unclear at this point. Schiff (2010) was able to demonstrate model-based control of DBS using the simple neuron implementation of Rubin and Terman (2004). Although computationally cheaper than the full conductance based models, this still suffers from the problems discussed above. A logical next step in this work will be to show that the simple hybrid neuron can also be effective in model-based control strategies of DBS.

This concept may also prove efficacious in brain computer interfaces (BCI). Rather than contributing to the dynamic changes in brain dynamics, BCI applications would be used in estimating state and decoding measurements. This is a concept that, although promising, has proven difficult to achieve (Schiff, 2012). Low-power realizations of these systems, as suggested here, offer a cost-effective option as BCI theories mature.

Finally, the most important point on the study of neural control engineering is that often the best model is not the most physiological one, but the one that best reduces error (Schiff, 2012). This is important because focusing too much on model adequacy may take away from the more important task of producing better therapies. An important question that will need to be answered in this case is, how detailed does a BG network model need to be in order to prove effective in estimating pathological conditions? The next step in this work is to begin developing strategies based on the these models and the control theoretic approaches of Voss et al. (2004) and Schiff (2010, 2012).

Ultimately, until models are capable of predicting therapeutic outcomes, either through realistic biological results or through a dimensionality reduced interpretations, the pathological BG models will remain just a complement to physiological experiments.

## 5. Conclusions

The networks utilizing the simple hybrid neuron presented here may offer a mechanism for revealing mathematical details of BG function and dysfunction that are hidden by the complexity of other models. An immediate extension that highlights that concept is in the parameter exploration of the RT model. The computational efficiency of the network presented in section 2.4 has allowed us to begin exploring the parameter space using a commodity computing cluster. Sweeps can be completed in hours as opposed to months of computing it would take to explore the original RT Model. We hope to present details of this in future publications.

Although we have chosen the Izhikevich hybrid neuron, there are other neuron models that could have been employed. The most obvious choice is the adaptive exponential integrate-and-fire neuron (Brette and Gerstner, 2005). Given the similarities of the two models we would predict the existence of parameter sets that would provide similar results. Rate or the population based models may also be an option. These, as well as the feasibility of their hardware implementations, are options that should be explored in the future.

Using the simple hybrid neuron, or any point neuron model, in such small networks and deriving biologically significant meaning from them can be unreliable. Care must be taken when interpreting the results in the context of both pathological conditions as well as clinical therapies. The traditional niche for the simple hybrid neuron has really been in large-scale modeling. The more biologically realistic conductance based neuron models are generally recommended for single and small-scale network studies (Izhikevich, 2007). In addition to those presented above, one of the primary motivations for using the simple model lie in the intention to construct large-scale models of the BG. This work presents the foundations for those future studies and the results demonstrate that the hybrid model is capable of capturing many of the relevant BG responses and dynamics. These studies are meant to complement experimental research as well as the more detailed modeling efforts.

### Conflict of interest statement

The authors declare that the research was conducted in the absence of any commercial or financial relationships that could be construed as a potential conflict of interest.
